# Harnessing the power of virtual (digital) twins: Graphical causal tools for understanding patient and hospital differences

**DOI:** 10.1016/j.csbj.2025.08.017

**Published:** 2025-08-27

**Authors:** Hemant Ishwaran, Eugene H. Blackstone

**Affiliations:** a Division of Biostatistics, Miller School of Medicine, University of Miami, Miami, USA; b Department of Quantitative Health Sciences, Cleveland Clinic, Cleveland, OH, USA; c Department of Thoracic and Cardiovascular Surgery, Cleveland Clinic, Cleveland, OH, USA

**Keywords:** Anomaly score, Bad virtual (digital) twin, Casual estimate, Relative risk, Standardized *β*, Variable selection

## Abstract

Traditional methods for evaluating hospital performance, such as regression or propensity score analysis, offer population-level comparisons but lack the granularity required for patient-level insight. We propose a causal framework based on virtual (digital) twins, enabling counterfactual outcome comparisons for individual patients across hospitals. Using data from the American Association for Thoracic Surgery (AATS) Quality Gateway Adult Cardiac Database, which includes 52,792 surgeries across 19 hospitals, we estimate patient-level causal effects for adverse surgical outcomes. Our approach combines model-free variable priority screening, random forests quantile classification (RFQ) for handling rare events, and isolation forests to assess treatment overlap and exclude invalid counterfactuals. Building on prior work, we introduce graphical tools for overlap diagnostics and counterfactual visualization at both the institutional and patient level. These tools reframe outcome modeling as individualized causal inference and support transparent, patient-centered hospital benchmarking.

## Graphical abstract

**Figure:**
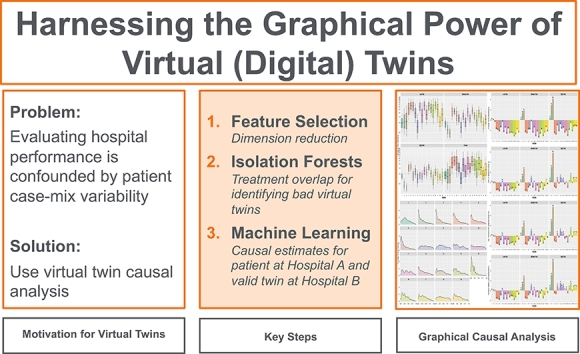

## 1 Introduction

Hospital performance is typically assessed using ranking systems, such as the U.S. News Best Hospitals Honor Roll [9], and star-based ratings, which are employed by organizations such as the Society of Thoracic Surgeons (STS), the Centers for Medicare and Medicaid Services, and, more broadly, by consumer platforms for rating services [7]. While these systems can incentivize quality improvement, they often lack the granularity needed for real-time monitoring and individualized evaluation. Within hospitals, quality officers frequently rely on unadjusted outcome summaries and Mortality and Morbidity (M&M) conferences [13], which, though informative, remain largely qualitative and lack a systematic framework for patient-focused comparisons.

To address these limitations, the American Association for Thoracic Surgery (AATS) introduced the AATS Quality Gateway (AQG) [1], an initiative that integrates statistical and machine learning methods into hospital quality assessment. Our study extends this effort by introducing a data-driven framework that supports patient level counterfactual comparisons across hospitals, enabling individualized benchmarking beyond traditional aggregate approaches.

Achieving this level of individualized assessment requires robust methods for causal inference using observational data. Propensity score methods [19], [21] have been widely used to mitigate selection bias and facilitate comparisons between treatment groups. These approaches typically estimate average treatment effects using matched or weighted groups, but often fall short when the goal is to simulate outcomes for specific patients or when treatment assignment depends on complex, high-dimensional covariates.

To overcome the limitations of traditional population-level methods and enable individualized causal inference, we adopt the virtual (or digital) twins framework, which simulates alternative outcomes for specific patients. The term “virtual twin”, introduced by [6], was originally developed to estimate individual treatment effects from clinical trial data. More recently, the related term “digital twin” has gained prominence in biomedical contexts [8], [4], [15], [12]. Both refer to the same underlying concept: creating a synthetic copy of a patient to simulate what might happen under a different exposure. While most applications compare treatment *A* versus treatment *B*, the framework naturally generalizes to our setting of comparing outcomes across hospitals. In our formulation, a patient treated at one hospital (which we can think of as treatment *A*) is virtually transferred to another (treatment *B*) by estimating their expected outcome using that hospital's predictive model. This allows us to ask: *What would have happened if this patient had been treated at a different hospital under similar conditions?*

To operationalize this framework, our analysis draws on a dataset comprising 52,792 adult cardiac surgeries performed between July 2017 and January 2020 across 19 hospitals from three high-performing systems participating in the AATS Quality Gateway initiative [10]. The data encompass a wide range of procedures, including coronary artery bypass grafting, valve surgeries, and thoracic aortic interventions. Outcomes of interest, previously modeled in related work [10], [23], include operative mortality, stroke, deep sternal wound infection, renal failure, prolonged ventilation, cardiac reoperation, prolonged length of stay, and a composite of major morbidity or mortality. In that earlier work, machine learning techniques such as random forests quantile classification [18] and model-free variable screening [16] were used to develop accurate risk models for outcome prediction and risk stratification.

In this study, we extend these models by enabling individualized benchmarking through estimates of how a patient's outcome might have differed had they received care at another hospital. Building on the virtual twins framework, our contribution lies in assembling a practical system that integrates machine learning, virtual twin causal inference, and diagnostic tools for hospital-level performance evaluation. We introduce graphical tools for overlap assessment and counterfactual visualization, including hospital effect plots, isolation forests to detect regions of poor overlap, and diagnostics to flag patients lacking valid comparators. Together, these components enable individualized benchmarking and offer a transparent framework for understanding institutional variability in surgical outcomes.

## 2 Methods and materials

A non-technical description of our approach is as follows. The goal is to compare outcomes between hospitals in a way that mimics a controlled experiment. Ideally, we would observe each patient receiving treatment at multiple hospitals to directly measure differences in outcomes. Since this is not possible, we use machine learning to create “virtual twins” counterfactual predictions of what a patient's outcome would have been had they been treated at a different hospital. By comparing a patient's actual outcome to their predicted counterfactual outcome, we estimate the causal effect of hospital treatment. This approach enables fair, patient-specific comparisons across institutions.

To implement our framework, we train machine learning models separately for each hospital. Specifically, treatment *A* denotes the hospital under study, while treatment *B* serves as a comparator. Each hospital's model predicts outcomes under its care, enabling counterfactual estimation across hospitals. While any machine learning method could be used in principle, we adopt random forests (RF) [3] due to their flexibility, ability to capture complex interactions, and built-in mechanism for out-of-bag (OOB) prediction [2], which reduces overfitting and improves generalizability.

A key challenge in this setting is class imbalance: most outcomes in the AQG database are binary adverse events, such as operative mortality, stroke, or renal failure, that occur infrequently [10], [23]. To address this, we employ random forest quantile classification (RFQ) [18], a modification of RF specifically designed for imbalanced outcomes. RFQ satisfies a dual optimality criterion and yields well-calibrated probability estimates without the need for external adjustment, making it especially well suited to rare event prediction in surgical outcomes.

Using RFQ, data from Hospital *A* is used to develop a predictive outcome model specific to *A*, while a separate RFQ model is trained on data from Hospital *B* to obtain a predictive outcome model specific to *B*, with both models using the same dependent variables. Under the assumptions of strong unconfoundedness and treatment overlap, unbiased estimates of causal effect sizes can then be obtained. These assumptions, collectively known as strongly ignorable treatment assignment [19], ensure valid causal inference. A more detailed discussion of these assumptions and our causal estimation procedure is provided below.

### 2.1 Strong unconfoundedness

In causal inference, strong unconfoundedness, as defined by Rubin [20], ensures the validity of causal effect estimation from observational data by assuming that treatment assignment is independent of potential outcomes, conditional on observed covariates.

To formally define this, we use (X,Y) to denote the covariate feature vector and *Y* for the binary outcome response. Let *T* denote the treatment assignment, where T=A represents Hospital *A*, and T=B represents Hospital *B*. Let Y(A) and Y(B) be the potential outcomes under treatments *A* and *B*, respectively. These represent the two possible outcomes a patient would have experienced if treated at either hospital. While a patient's outcome is only observed under their actual treatment assignment, the other potential outcome remains unobserved and can be conceptually regarded as the patient's virtual twin.

The assumption of strong unconfoundedness states that:

{Y(A),Y(B)}⊥T|X.

This assumption implies that, conditional on **X**, the treatment assignment *T* is independent of the potential outcomes. In other words, after adjusting for all relevant confounding variables **X**, the assignment to Hospital *A* or Hospital *B* is effectively randomized, enabling causal effect estimation from observational data.

While strong unconfoundedness is inherently untestable, it is more likely to hold when many independent predictors are measured and known to be informative for the outcome. The informativeness of predictors can be evaluated using variable selection techniques, such as model-free variable priority screening [16], which is also employed for assessing treatment overlap, discussed next. Previous work [10], [23] identified a comprehensive set of predictive variables for surgical outcomes in our database, providing empirical evidence supporting the plausibility of the strong unconfoundedness assumption.

### 2.2 Treatment overlap (the positivity condition)

The assumption of treatment overlap, also referred to as the positivity condition in causal inference, ensures that each patient has a nonzero probability of receiving either treatment *A* or *B*, given their covariates. This assumption prevents issues where certain covariate values are exclusive to only one treatment group, which would make causal effect estimation impossible for those subpopulations.

Formally, treatment overlap requires that the probability of receiving each treatment, known as the propensity score, satisfies:

0<P(T=A|X)<1for all X.

Equivalently, this can be expressed as:

0<e(X)<1,where e(X)=P(T=A|X)

denotes the propensity score. This condition ensures that for every covariate profile **X**, there exist patients treated at both Hospital *A* and Hospital *B*. Without treatment overlap, there would be regions in the covariate space where only one treatment is observed, making it impossible to estimate counterfactual outcomes for patients in those regions.

Treatment overlap must be assessed for each patient. Traditionally, this is evaluated using propensity score diagnostics, such as overlap plots or standardized mean differences, or by fitting regression models, such as logistic regression, with treatment assignment as the dependent variable. However, we adopt a more robust, data-driven approach using isolation forests [14], an unsupervised anomaly detection method. In isolation forests, anomalies are identified by randomly partitioning data and detecting outliers based on tree split depth. Observations with shorter path lengths are considered outliers, as they require fewer splits to become isolated. A threshold, such as the 5th percentile of path lengths, is used to flag anomalous cases. To enhance robustness, this process is repeated across multiple trees, generating a forest anomaly score.

In more detail, the verification of treatment overlap using isolation forests proceeds as follows. A key step is the selection of independent variables that are predictive of the adverse outcome under study. This ensures that the anomaly score is trained on features directly relevant to the causal effect of interest, enhancing its ability to detect violations of overlap.

1. Use model-free variable priority screening [16] to identify variables most predictive of the outcome. These variables form the input feature set for evaluating overlap.
2. Train an isolation forest on hospital *A*'s data using the variables from Step 1. The model is built in an unsupervised manner, without using outcome values, to capture the distribution of covariates in hospital *A*.
3. Apply the trained isolation forest to patients from hospital *B* to compute anomaly scores. Patients with scores below a threshold percentile of the hospital *A* distribution are flagged as “bad virtual twins” indicating they are too dissimilar from hospital *A* patients to reasonably assume overlap. These patients are excluded from the causal comparison between hospital *A* and *B*.

### 2.3 Causal estimates

Causal estimates are defined as the predicted value from the outcome model *A* compared with the predicted value from the outcome model *B* for a patient from *B* that has an appropriate virtual twin in *A*. We can describe this more formally using the following mathematical notation.

Let $L=\{(x_{i},y_{i}):i=1,\ldots ,n\}$ denote the training data, where $x_{i}$ is the patient's observed *p*-dimensional feature vector and $y_{i}\in \{0,1\}$ is the observed adverse binary outcome. For a specific hospital h∈{1,…,H}, we denote its training data as $L_{h}=\{(x_{i}^{\left(h\right)},y_{i}^{\left(h\right)}):i=1,\ldots ,n^{\left(h\right)}\}$. In our database, there are H=19 hospitals, with a feature dimension of p=367. The total sample size is n=52,792, distributed across the hospitals such that $n=\sum_{h}n^{\left(h\right)}$.

We are interested in evaluating the performance of hospital *h* relative to all hospitals in the system. Here, hospital *h* corresponds to Hospital *A* described earlier. Performance is assessed using the overall hospital O={1,…,H}, which represents the full dataset, including hospital *h* itself, and corresponds to the previously defined Hospital *B*. Since the analysis is repeated for each hospital *h*, defining the overall hospital in this way ensures that every hospital is evaluated against a common and comprehensive reference population, thus allowing for a fair and consistent assessment of hospital performance.

#### 2.3.1 True risk

The true risk for a hypothetical patient with covariate values **x**, if treated at hospital *h*, is defined as:

$$\theta_{h}(x)=P_{h}\{Y=1|X=x\}$$

while the true risk for the same hypothetical patient, if treated at the overall hospital *O*, is defined as:

$$\theta_{O}(x)=P_{O}\{Y=1|X=x\}.$$

The causal effect for **x** is then defined as:

$$\tau_{h}(x)=\frac{\theta_{h}(x)}{\theta_{O}(x)}$$

representing the causal relative risk, where values less than one indicate a better outcome if treated at hospital *h* compared to the overall hospital performance.

#### 2.3.2 Random forests estimated risk

Since these true risks are unknown, they must be estimated from data. The hospital-specific risk predictors for *h* and *O* are obtained using RFQ trained on different datasets. The predictor for hospital *h*, denoted ${\overset{ˆ}{\theta }}_{h}(\cdot )$, is estimated from a random forest trained on hospital *h*'s data $L_{h}$. Similarly, the overall hospital predictor ${\overset{ˆ}{\theta }}_{O}(\cdot )$ is estimated using a random forest trained on the entire dataset L.

For a hypothetical patient **x**, these estimators provide counterfactual risk predictions: ${\overset{ˆ}{\theta }}_{h}(x)$ represents the estimated risk if the patient **x** were treated at hospital *h*, while ${\overset{ˆ}{\theta }}_{O}(x)$ provides the estimated risk under the overall hospital model. The causal effect for **x** is then estimated as:

$${\overset{ˆ}{\tau }}_{h}(x)=\frac{{\overset{ˆ}{\theta }}_{h}(x)}{{\overset{ˆ}{\theta }}_{O}(x)}.$$

It is also convenient to work with the log relative risk:

$$\log({\overset{ˆ}{\tau }}_{h}(x))=\log({\overset{ˆ}{\theta }}_{h}(x))-\log({\overset{ˆ}{\theta }}_{O}(x))$$

where values less than zero indicate that patient **x** fares better at hospital *h* on average.

#### 2.3.3 Random forests OOB estimated risk

The previous discussion considered a hypothetical patient **x**, but in practice, causal effects are provided for the training cases. To prevent overfitting on this data, we use the out-of-bag (OOB) random forest predictor for constructing these estimates. The OOB predictor is constructed by aggregating predictions from trees where a given training point was not used in the bootstrap sample. Since each tree is trained on a random subset of the data, approximately 37% of the observations are excluded from each bootstrap sample and serve as OOB data. By averaging predictions across these excluded trees, the OOB predictor provides an estimate that is independent of the training point itself. This construction is a generalization of the leave-one-out cross-validation framework [5], making it a robust method for reducing overfitting while maintaining predictive accuracy.

Denoting the OOB predictor by ${\overset{ˆ}{\theta }}_{h}^{⁎}(\cdot )$ for hospital *h* and ${\overset{ˆ}{\theta }}_{O}^{⁎}(\cdot )$ for the overall hospital, the causal effect for a training data point $x_{i}^{\left(h\right)}$ from hospital *h* is estimated as:

(1) $${\overset{ˆ}{\tau }}_{h}^{⁎}(x_{i}^{\left(h\right)})=\frac{{\overset{ˆ}{\theta }}_{h}^{⁎}(x_{i}^{\left(h\right)})}{{\overset{ˆ}{\theta }}_{O}^{⁎}(x_{i}^{\left(h\right)})},\;\log({\overset{ˆ}{\tau }}_{h}^{⁎}(x_{i}^{\left(h\right)}))=\log({\overset{ˆ}{\theta }}_{h}^{⁎}(x_{i}^{\left(h\right)}))-\log({\overset{ˆ}{\theta }}_{O}^{⁎}(x_{i}^{\left(h\right)}))$$

On the other hand, for a training data point $x_{i}^{\left(-h\right)}$ from outside hospital *h*, the causal effect is estimated as:

(2) $${\overset{ˆ}{\tau }}_{h}^{⁎}(x_{i}^{\left(-h\right)})=\frac{{\overset{ˆ}{\theta }}_{h}(x_{i}^{\left(-h\right)})}{{\overset{ˆ}{\theta }}_{O}^{⁎}(x_{i}^{\left(-h\right)})},\;\log({\overset{ˆ}{\tau }}_{h}^{⁎}(x_{i}^{\left(-h\right)}))=\log({\overset{ˆ}{\theta }}_{h}(x_{i}^{\left(-h\right)}))-\log({\overset{ˆ}{\theta }}_{O}^{⁎}(x_{i}^{\left(-h\right)})).$$

Since $x_{i}^{\left(-h\right)}$ is not part of hospital *h*'s training data, it is treated as an external test point for ${\overset{ˆ}{\theta }}_{h}$, meaning the full sample trained estimator is used rather than the OOB procedure.

#### 2.3.4 Eliminating bad virtual twins: final estimated values

To be in compliance with treatment overlap, we only utilize causal estimates from comparable virtual twins. In order to eliminate poor matches, we use an isolation forest to compute an anomaly score that quantifies the similarity of each observation to the training distribution for a given hospital. Let $i_{h}(\cdot )$ be the anomaly score for hospital *h*, trained using its data $L_{h}$. These scores are calibrated on the unit interval, with lower values indicating greater deviation from the core structure of the training data and hence poorer support.

We exclude causal estimates for which either the observed or virtual twin falls in a low-density region of the corresponding hospital's training distribution. Specifically, estimators (1) and (2) are used only when $i_{h}(x_{i}^{\left(h\right)})<C$ and $i_{h}(x_{i}^{\left(-h\right)})<C$, where *C* is the anomaly score threshold. In our analysis, we set C=0.05, which corresponds to excluding the 5 percent most anomalous observations as measured by the training distribution for each hospital. This value was chosen as a conservative heuristic to reduce extrapolation risk in low-support regions, thereby improving reliability of the causal estimates. We emphasize that *C* is a tunable parameter, and its value can be modified in future applications or subjected to sensitivity analysis as needed.

Thus, for hospital *h*, we obtain causal relative risk values (similarly log relative-risk values)

(3) $$\left\{{\overset{ˆ}{\tau }}_{h}^{⁎}(x_{i}^{\left(h\right)}):i_{h}(x_{i}^{\left(h\right)})<C\},\;\{{\overset{ˆ}{\tau }}_{h}^{⁎}(x_{i}^{\left(-h\right)}):i_{h}(x_{i}^{\left(-h\right)})<C\right\}$$

for the training data having compliant virtual twins. There can be a maximum of *n* such values, but typically this value is smaller and will depend on the cutoff *C*.

### 2.4 Statistical analysis

Variable selection using model-free variable priority screening [16] was implemented with the R-package varPro, available at https://github.com/kogalur/varPro. The package also provides customized isolation forest modeling which were employed for the treatment overlap analysis. Random forest models, including RFQ, used for causal estimates were performed using the CRAN R-package randomForestSRC [11].

## 3 Results

Among the various adverse outcomes available in the data, we focus on operative mortality, a critical outcome of particular interest to AQG. This choice ensures a concrete and clinically meaningful analysis, reflecting the focus of prior work [10], [23]. Below, we present the results of this analysis, organized into subsections based on the granularity of the findings.

### 3.1 Overall summary analysis

**Fig. 1:**
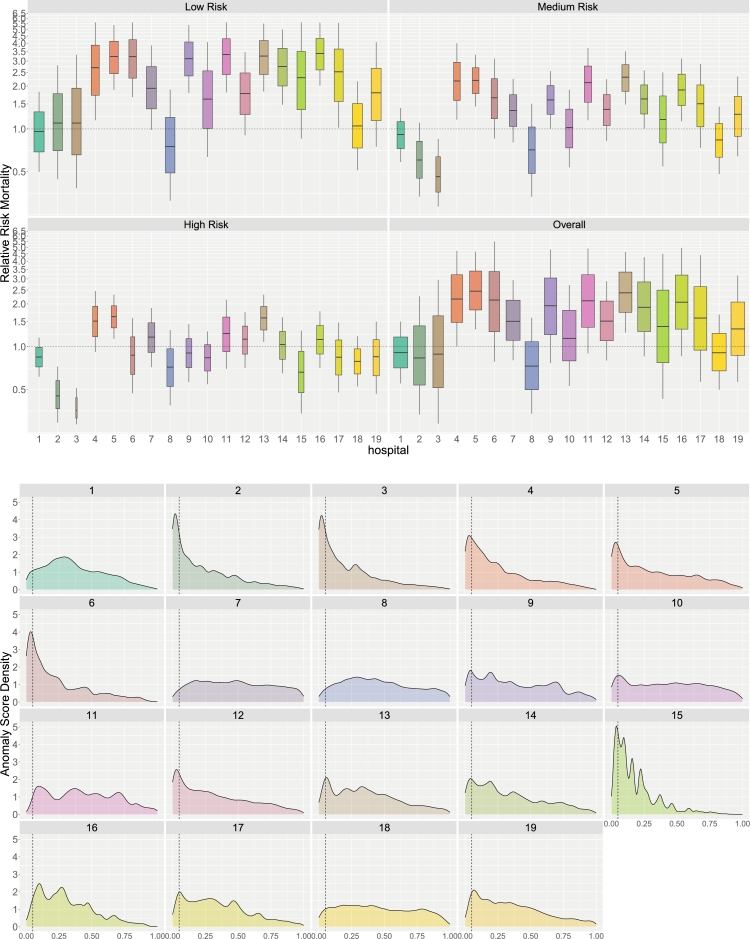
Causal relative risk estimates and isolation forest anomaly scores for each hospital. The top figures present causal relative risk estimates, stratified by overall risk and displayed as boxplots scaled to hospital size (wider boxplots indicate larger hospitals). Relative risk is categorized into percentiles: 0-50 (low risk), 50-75 (medium risk), and 75-100 (high risk), with “Overall” representing unstratified values. A symmetrical logarithmic scale ensures that values below 1.0 indicate a hospital outperforming the average. The bottom figures display the density of isolation forest anomaly scores, where lower values suggest a lower probability of a valid virtual twin. The thin vertical line at 0.05 is the cutoff *C* used to screen bad virtual twins.

Summary results are provided conveniently in graphical format given in Fig. 1. The top set of figures displays causal relative risk estimates (3) for each hospital, stratified by overall risk, with boxplot widths scaled to the sample size. Using the overall hospital *O* as a baseline, patient risk values were determined and grouped into percentiles: 0-50 (low risk), 50-75 (medium risk), and 75-100 (high risk). The “Overall” category represents unstratified relative risk values.

The relative risk plots, akin to forest plots in meta-analyses but rotated 90 degrees, use a symmetrical logarithmic scale. The distance from 1.0 to 2.0 matches that from 1.0 to 0.5, and similarly, the distance from 1.0 to 4.0 matches that from 1.0 to 0.25. This ensures an equivalent interpretation above and below 1.0: values below 1.0 indicate a hospital outperforming the average. We observe that hospitals h=2,3 generally outperform most hospitals, with their advantage increasing for high-risk patients. Overall, these hospitals demonstrate superior performance.

The bottom set of figures displays the estimated density of the isolation forest anomaly scores $\left\{i_{h}(x_{i}):i=1,\ldots ,n\right\}$ for each hospital *h*. Smaller values indicate a lower probability of a valid virtual twin, highlighting potential violations of the treatment overlap assumption. Hospital 15 exemplifies this, showing a highly left-skewed density, suggesting a substantial number of poor matches for the case study. In contrast, hospital h=1 serves as a good match, as its density remains low over small anomaly scores, indicating better treatment overlap and comparability for most of its patients.

### 3.2 Performance for select patient features

**Fig. 2:**
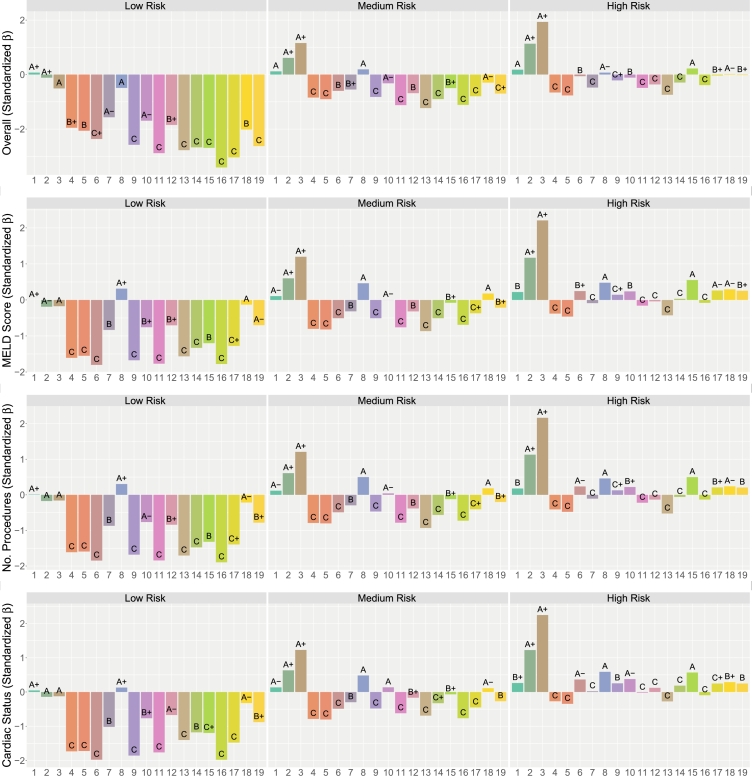
Standardized *β* values from an ANOVA for MELD Score, Number of Cardiac Procedures, and Cardiac Status, with standardized log relative risk as the dependent variable. Estimates are stratified by overall risk, and hospitals are assigned letter grades (A+, A, A-, B+, B, B-, C+, C) based on their percentile ranking within each stratification. A positive *β* indicates superior performance relative to the baseline. Hospitals *h* = 2,3 consistently receive the highest grades, with significantly larger positive *β* values both overall and across the three independent variables.

To further investigate differences between hospitals, Fig. 2 presents standardized *β* values from an ANOVA. The analysis focuses on the top three variables identified in the analysis filtering step: MELD Score (Model for End-Stage Liver Disease), Number of Cardiac Procedures, and Cardiac Status. The standardized log relative risk, defined as the log relative risk divided by the overall sample standard deviation, was used as the dependent variable. An ANOVA model was fit with terms for the independent variable and hospital-specific intercepts. For the “Overall” plot, only hospital intercepts were included, without an independent variable. The displayed standardized *β* values represent the estimated intercepts, providing insight into hospital-specific effects.

The standardized *β* values are stratified by risk, similar to the previous figure. Within each panel, hospitals are assigned a traditional letter grade, ranging from high (A+, A, A-) to lower grades (B-, C+, C), based on the percentile ranking of a hospital's *β* value relative to other hospitals within the same risk stratification. Thus, a grade of A in the “Overall” low-risk group indicates hospitals that perform above average within that group. A positive *β* is calibrated to indicate superior performance relative to the baseline. However, a hospital can receive a high grade, such as A-, even if its *β* value is near zero or slightly negative, as grades are assigned based on relative rankings rather than absolute values.

As before, hospitals h=2,3 consistently receive the highest letter grades. Their standardized *β* values are significantly larger and more positive than those of other hospitals, both overall and across all three independent variables.

### 3.3 Performance across a spectrum of features for finer resolution

**Fig. 3:**
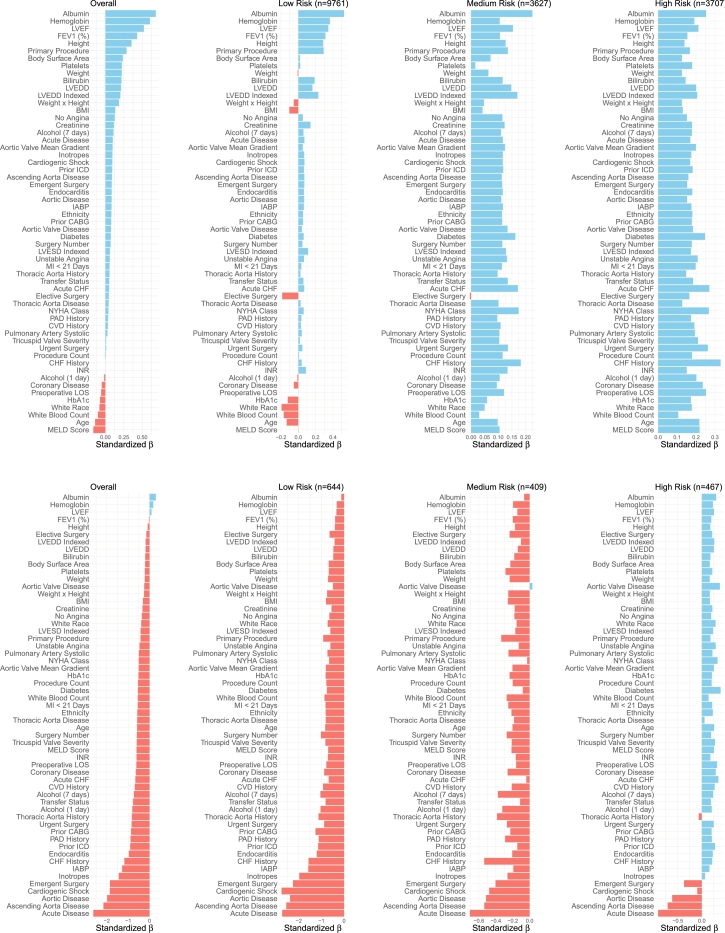
Standardized *β* values for hospital *h* = 1 (top) and *h* = 19 (bottom) across all variables identified in the feature screening. Hospital *h* = 1, a large hospital with good performance, has uniformly positive *β* values, with particularly high values for high-risk patients. In contrast, the smaller hospital *h* = 19 shows predominantly negative *β* values (red) across groups, except for high-risk patients, where they are mostly positive (blue), reflecting its relatively better performance in this subgroup.

To examine hospital performance at a finer patient level, Fig. 3 presents standardized *β* values for selected hospitals using all independent variables identified from the analysis filtering step. The top panel displays values for hospital h=1, while the bottom panel shows values for hospital h=19.

Hospital h=1, a good performer with a large sample size, is immediately recognizable as all its *β* values are positive, with particularly large values for high-risk patients. In contrast, hospital h=19, with a smaller sample size, exhibits a different pattern. From the top panel of Fig. 1, we observe that while its overall performance is slightly worse than the average hospital, it performs slightly better for high-risk patients. This trend is mirrored in the bottom panel of Fig. 3, where nearly all standardized *β* values are negative (red) across groups, except for high-risk patients, where they are mostly positive (blue).

### 3.4 Subgroup treatment strategies

Hospitals h=2,3 were identified as high-performing institutions in our analysis. Given their superior outcomes, we further examine their relative performance in managing high-risk patients—those who stand to benefit the most from treatment at a hospital equipped to handle complex cases. Understanding how these hospitals perform for such patients can provide critical insights into optimizing surgical care.

To investigate this, we focused on patients who underwent isolated aortic valve replacement with more than one planned cardiac procedure. This group represents a significantly high-risk population due to their complex comorbidities, increased surgical burden, and heightened risk of postoperative complications. Identifying the optimal hospital for these patients could lead to substantial improvements in surgical outcomes and overall survival.

Further restricting the analysis to those with comparable virtual twins, we applied an ANOVA model with standardized log relative risk as the dependent variable. Unlike our previous analysis, which compares standardized log relative risk for a hospital against the overall average, this approach evaluates the log relative risk between the two hospitals:

$$\log(\tau_{\begin{array}{c} 2∼3 \end{array}}(x)):=\log(\theta_{3}(x))-\log(\theta_{2}(x)).$$

The *β* coefficient is calibrated such that a positive value indicates better mortality outcomes for hospital h=2 compared to h=3, while a negative value indicates better outcomes for hospital h=3.

**Fig. 4:**
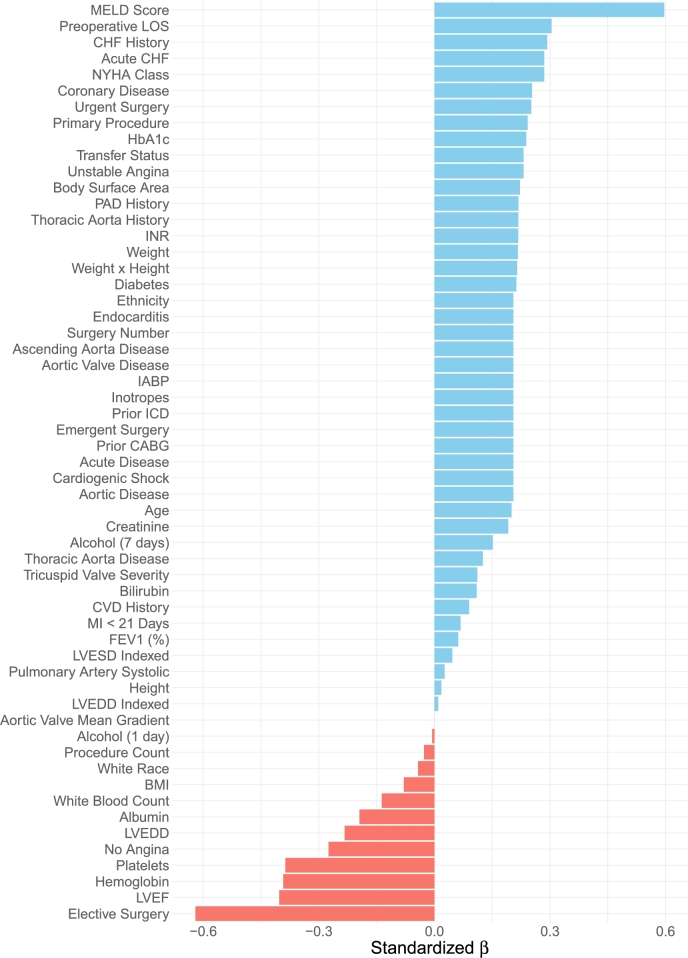
Standardized *β* values from ANOVA modeling of high-risk patients undergoing isolated aortic valve replacement with multiple planned cardiac procedures. The *β* coefficients are sorted from largest positive to largest negative, where positive values (blue) indicate better mortality outcomes at hospital *h* = 2, and negative values (red) indicate better outcomes at hospital *h* = 3. Overall, hospital *h* = 2 demonstrates superior performance for this subgroup, though certain patients exhibit better outcomes at hospital *h* = 3.

Fig. 4 displays the standardized *β* values from the ANOVA, with coefficients sorted from the largest positive to the largest negative for the same variables as in Fig. 3. A clear pattern emerges: hospital h=2 generally outperforms hospital h=3 for this specific population, as indicated by the greater number of positive coefficients (displayed in blue). However, several variables exhibit large negative coefficients (displayed in red), suggesting that certain patient subgroups may experience better outcomes at hospital h=3.

Key patient characteristics influencing management strategies can be identified by examining variables with large coefficient magnitudes (these being variables near the top and bottom of the figure). One such variable is the preoperative MELD score for a patient and which was previously identified as a top predictor in the analysis filtering step. Originally developed to assess chronic liver disease severity and predict survival in liver transplantation, the MELD score [17] is also a strong predictor of postoperative complications in cardiac surgery, including renal failure, prolonged ICU stay, and in-hospital mortality. The large positive coefficient suggests that patients with higher MELD scores fare significantly better at hospital h=2, possibly due to superior management of comorbidities or perioperative care.

Another example is whether the patient opted for elective surgery. In this case, we observe a large negative *β* value for this variable, indicating that elective surgery patients experienced higher mortality at hospital h=2. This suggests that hospital h=3 may have superior protocols or preoperative management strategies for elective procedures, leading to improved outcomes for these patients.

## 4 Discussion

A key challenge in hospital comparisons is case-mix variability. Traditional risk-adjusted outcomes assess a provider's performance relative to an average provider but do not ensure that hospitals treat comparable patients. Recognizing this, STS reports ratings rather than rankings to avoid misleading comparisons [22]. Our virtual twins framework addresses this concern directly by generating patient-specific counterfactual predictions, allowing each patient to be compared only to their virtual twin, an alternative version of themselves treated at a different hospital. This ensures that hospitals are evaluated under matched case-mix conditions, reducing bias from institutional differences.

In addition to improving fairness in benchmarking, virtual twins fit within the broader goals of precision medicine. Because our approach relies on predicted outcomes rather than observed adverse event rates, it is less sensitive to small-sample noise and case-mix confounding. Comparisons are restricted to patients with valid matches, further enhancing interpretability and robustness. At the point of care, these individualized estimates could inform patient and surgeon decision-making by identifying hospitals with more favorable predicted outcomes for specific profiles. At the system level, risk-adjusted performance can be stratified by procedure type, patient subgroup, or institutional characteristics to guide resource allocation and quality improvement. Hospitals can also benchmark key risk factors against peers to identify strategic priorities.

As with all observational studies, our framework relies on assumptions that are necessary for valid causal inference but cannot be directly verified from the data. Chief among these is the assumption of strong unconfoundedness, which requires that all variables influencing both treatment assignment and outcomes are adequately measured and included in the models. Although our database contains a rich set of preoperative and intraoperative features previously shown to be predictive of surgical outcomes [10], [23], unmeasured confounding remains a potential source of bias. In particular, institutional factors such as staffing, care coordination, or postoperative protocols, though partially captured through hospital identifiers, may not be fully accounted for through patient-level covariates alone. Likewise, our approach assumes adequate treatment overlap, meaning that patients from different hospitals share sufficiently similar covariate profiles to allow valid counterfactual comparisons. While we employed diagnostic tools, including variable importance screening and isolation forest analysis, to assess and filter poor overlap cases, the possibility of residual extrapolation in low-density regions cannot be ruled out. As such, our results should be interpreted as estimates of causal effects under the assumption of no unmeasured confounding and adequate overlap, supported but not guaranteed by the structure of the data.

While large-scale implementation presents challenges, AQG is actively working to embed virtual twins into its analytics pipeline. This will allow the methodology to become a core component of modern hospital quality assessment, promoting more transparent and patient-centered evaluation across healthcare systems.

## 5 Conclusion

This study introduces a data-driven, patient-centered framework for evaluating hospital performance using virtual twins methodology. By integrating machine learning and causal inference techniques, our approach enables individualized counterfactual comparisons, moving beyond traditional risk-adjusted benchmarking. Virtual twins ensure equitable hospital comparisons by mitigating biases from case-mix differences, providing a more transparent and interpretable assessment of hospital performance.

Our methods incorporate random forest quantile classification to address class imbalance in rare surgical outcomes and isolation forests to assess treatment overlap, ensuring robust causal effect estimation. The framework is further enhanced by visualization tools that support both high-level hospital assessments and granular patient-level insights.

Future work will refine predictive modeling, incorporate additional clinical and institutional factors, and expand applications to broader healthcare quality assessments. Although we focus here on hospital-level comparisons, the same framework is readily extensible to alternative exposures such as treatment strategies or provider types. In principle, the structure we describe can accommodate any categorical exposure, whether hospitals, treatments, providers, or care protocols, provided that overlap and covariate support conditions are met. Enhanced interactive visualization tools will improve accessibility and usability for stakeholders.

## CRediT authorship contribution statement

**Hemant Ishwaran:** Writing – review & editing, Writing – original draft, Conceptualization. **Eugene H. Blackstone:** Writing – review & editing, Conceptualization.

## Declaration of Competing Interest

The authors, Hemant Ishwaran and Eugene Blackstone, declare no conflicts of interest.

## Data Availability

Our code is publicly available as an R-package varPro, which can be accessed at the repository https://github.com/kogalur/varPro. Random forest models were performed using the open source CRAN R-package randomForestSRC [11].
